# Circulating tumour cells & circulating tumour DNA in patients with resectable colorectal liver metastases (MIRACLE): a prospective, observational biomarker study

**DOI:** 10.1016/j.eclinm.2025.103406

**Published:** 2025-08-12

**Authors:** Lissa Wullaert, Maurice P.H.M. Jansen, Jaco Kraan, Yannick M. Meyer, Kelly Voigt, Stavros Makrodimitris, Vanja de Weerd, Corine M. Beaufort, Mai Van, Maarten Vermaas, Eric J.T. Belt, Paul D. Gobardhan, Stefan Sleijfer, Henk M.W. Verheul, John W.M. Martens, Dirk J. Grünhagen, Saskia M. Wilting, Cornelis Verhoef, the MIRACLE study team, Ninos Ayez, Jan H. Wijsman, Arjen M. Rijken, Pascal Doornebosch, Joost van der Hoeven, Boris Galjart, Diederik J. Höppener, Peter M.H. Nierop, Eric P. van der Stok, Jean Helmijr, Lindsay Angus, Pauline A.J. Mendelaar, Manouk K. Bos, Elisabeth M. Jongbloed, Khrystany T. Isebia, Noortje Verschoor, Ronald van Marion, Peggy Atmodimedjo, Hendrikus J. Dubbink

**Affiliations:** a Department of Surgical Oncology and Gastrointestinal Surgery, Erasmus MC Cancer Institute, Erasmus University Medical Center, Rotterdam, the Netherlands; b Department of Medical Oncology, Erasmus MC Cancer Institute, Erasmus University Medical Center, Rotterdam, the Netherlands; c Department of Surgical Oncology and Gastrointestinal Surgery, IJsselland Hospital, Capelle aan den IJssel, the Netherlands; d Department of Surgical Oncology and Gastrointestinal Surgery, Albert Schweitzer Hospital, Dordrecht, the Netherlands; e Department of Surgical Oncology and Gastrointestinal Surgery, Amphia Hospital, Breda, the Netherlands

**Keywords:** CTC (circulating tumour cells), ctDNA (circulating tumour DNA), Liquid biopsy, Minimal residual disease (MRD), Colorectal liver metastases (CRLM)

## Abstract

**Background:**

Recurrence risk after curative surgery for colorectal liver metastases (CRLM) remains high, underlining the need to identify prognostic markers enabling more individualised treatment approaches.

**Methods:**

In the MIRACLE, a prospective, observational biomarker study, a total of 188 patients with isolated, resectable CRLM without (neo)adjuvant chemotherapy were included between October 2015 and December 2021. Blood samples were collected before surgery (baseline) and three weeks after surgery. The primary objective was to assess the potential association between postoperative circulating tumour DNA (ctDNA) detection and recurrence of disease for patients with resectable CRLM within one year after resection. The secondary objective was the association between recurrence of disease within one year and detection of circulating tumour cells (CTCs). Baseline ctDNA was measured by next generation sequencing using a targeted panel (Oncomine Colon cell-free DNA assay) and postoperatively by digital PCR on genetic variants found preoperatively with the Oncomine panel. CTCs were enumerated using the FDA-approved CellSearch system.

**Findings:**

ctDNA was detected in 117/187 patients (63%) at baseline, and 28/104 evaluable patients (27%) still had detectable ctDNA postoperatively. CTC enumeration resulted in positivity for 37/183 patients (20%) at baseline and 14/158 patients (9%) postoperatively. No association was found between 1-year recurrence-free survival (RFS) and the presence of CTCs or ctDNA at baseline. In contrast, patients with postoperative undetectable ctDNA had a significantly improved 1-year RFS compared to patients with postoperative ctDNA (54% [95% CI 44%–67%] vs. 25% [95% CI 13%–47%], log-rank p = 0.0011). Similarly, patients with postoperative detectable CTCs had a significantly shorter 1-year RFS compared to patients without postoperative CTCs (15% [95% CI 4%–55%] vs. 53% [95% CI 45%–62%], log-rank p 0.0004). Also in multivariable analysis, detectable ctDNA and CTCs after surgery remained independently associated with a shorter 1-year RFS (HR 2.35; 95% CI 1.34–4.11; p = 0.0028 and HR 2.98; 95% CI 1.56–5.71; p = 0.0010, respectively).

**Interpretation:**

This is the first study conducted in patients with resectable CRLM without (neo)adjuvant chemotherapy, which demonstrates the impact of postoperative detectable circulating tumour load on 1-year RFS. Postoperative ctDNA and CTC detection both represent strong, independent predictors for a shorter RFS after local treatment, as opposed to preoperative detection.

**Funding:**

This work was supported by 10.13039/501100004622KWF Kankerbestrijding (Dutch Cancer Society, EMCR 2014-6340).

**Research in context**

**Evidence before this study**

A comprehensive systematic search was conducted in electronic databases (Embase, Medline, Cochrane, Web of Science & Google Scholar) for studies published from database inception up to the 26th of May 2023. Studies investigating the association between circulating tumour DNA (ctDNA) and oncological outcomes in patients undergoing curative-intent local therapy for colorectal liver metastases (CRLM) were included. We found data from individual trials, mostly single institution, retrospective cohorts, suggesting associations between postoperative ctDNA and recurrence-free survival (RFS). However, sample sizes were small and heterogeneous treatment schemes including both pre- and/or post-operative chemotherapy were administered to patients, which potentially hampers interpretation of their results. We did not find studies focusing on only chemonaive patients and neither did we find studies with additional circulating tumour cell (CTC) analyses. Additional publications to date have not overcome these limitations or focused on RFS based on circulating tumour load unaffected by chemotherapy.

**Added value of this study**

In this cohort of resectable CRLM patients without any perioperative chemotherapy, detection of postoperative CTCs and ctDNA alone or combined were independent risk factors for a shorter 1-year RFS. To date, this study encompasses one of the largest cohorts of patients with resectable CRLM subjected to CTC and ctDNA analyses before and after local treatment. Notably, this is the first study to specifically investigate chemonaive, resectable CRLM patients, thereby ensuring a homogeneously treated cohort with circulating tumour load unaffected by any (neo)adjuvant chemotherapy treatment. Results show the impact of detectable circulating tumour load after surgery on RFS. Current risk score systems, like FONG for patients with CRLM, include solely preoperative clinical risk factors. Therefore, biomarkers like CTCs and ctDNA have the potential to provide insight into tumour bulk and may as such also be informative on the presence of remaining disease at several time points during treatment. Furthermore, the MIRACLE represents the only cohort that integrates the combination of ctDNA and CTC analyses in the clinical setting. Detection of at least one biomarker (CTCs or ctDNA) postoperatively was linked to a higher recurrence risk within the first year, highlighting the potential benefit of dual biomarker analysis in detecting all patients with a high risk for recurrence.

**Implications of all the available evidence**

The MIRACLE cohort shows that novel, real-time biomarkers reflecting microscopic disease, like ctDNA, can stratify patients into risk for recurrence. Just like in the primary setting, ctDNA may have the potential to guide clinicians and patients in perioperative therapy strategy decision-making. A recent subgroup analysis from the GALAXY trial by Katoaka et al. showed the potential benefit of adjuvant chemotherapy in RFS in a small cohort of patients with ctDNA detected postoperatively. Together these results strongly support the initiation of a randomised trial to determine the clinical benefit of ctDNA-guided adjuvant chemotherapy in this patient population. This could contribute to more personalised treatment strategies for patients with resectable CRLM and may translate into an overall survival benefit.

## Introduction

Approximately 25–50% of patients presenting with colorectal cancer (CRC) will be diagnosed with metastatic disease, simultaneously or after primary diagnosis, with the most common metastatic site being the liver.^1^^,^^2^ Among those with colorectal liver metastases (CRLM), about 19–25% of patients with synchronous CRLM and 50% of patients with metachronous CRLM have resectable disease and are eligible for curative treatment through local therapy^3^^,^^4^ However, around half of these patients are confronted with recurrence within one year after local treatment of CRLM.^5^ Perioperative chemotherapy may postpone recurrence, but randomised controlled trials have failed to demonstrate an overall survival (OS) benefit.^6^ This may be partly attributable to the underrepresentation of the group that could benefit from perioperative chemotherapy in these studies. If the specific patient subpopulation likely to benefit from additional chemotherapy could be accurately identified, future trials may be more successful in showing improved oncological outcomes.^7^

To identify patients that may profit from perioperative chemotherapy the detection of minimal residual disease (MRD) following surgery appears a promising approach.^8^^,^^9^ Current MRD approaches largely focus on the detection of cancer-derived material, such as circulating tumour cells (CTCs) or circulating tumour-derived DNA fragments in the blood of patients, which provides real-time information and can be measured repeatedly. CTCs are intact, viable tumour cells that are shed into the peripheral blood by the tumour and, when detected, have been shown to be of prognostic value in both the primary and metastasised setting.^10^^,^^11^ Circulating tumour DNA (ctDNA) is a tumour-derived component of the cell free DNA (cfDNA) found in the peripheral blood stream that is considered to be released through cancer cell apoptosis, necrosis and phagocytosis. Recent literature has shown that ctDNA is a prognostic marker in the setting of primary CRC and that ctDNA-guided treatment of stage II CRC is promising.^9^^,^^12^^,^^13^ Both CTCs and ctDNA have the potential to provide insight into tumour bulk and may as such also be informative on the presence of remaining disease at several time points during treatment.

Several studies have been conducted in the setting of CRLM in which a strong association was shown between detectable ctDNA after treatment and oncological outcomes.^14^, ^15^, ^16^ However, in these studies, heterogeneous treatment schemes including both pre- and postoperative chemotherapy were administered to patients, which hampers interpretation of their results. For example, chemotherapy has a substantial effect on circulating tumour load^13^^,^^14^ and therefore risk stratification for recurrence is challenging in patients with CRLM who have been locally treated in combination with perioperative chemotherapy. In order to work towards more personalised treatment, the MIRACLE study, a prospective and observational biomarker study in patients with resectable CRLM was conducted. The primary objective of this study was to assess the potential association between postoperative ctDNA detection and recurrence of disease for patients with resectable CRLM within one year after resection. As a secondary objective, the association between recurrence of disease within one year and detection of CTCs was also investigated.

## Methods

### Study design and participants

**Fig. 1:**
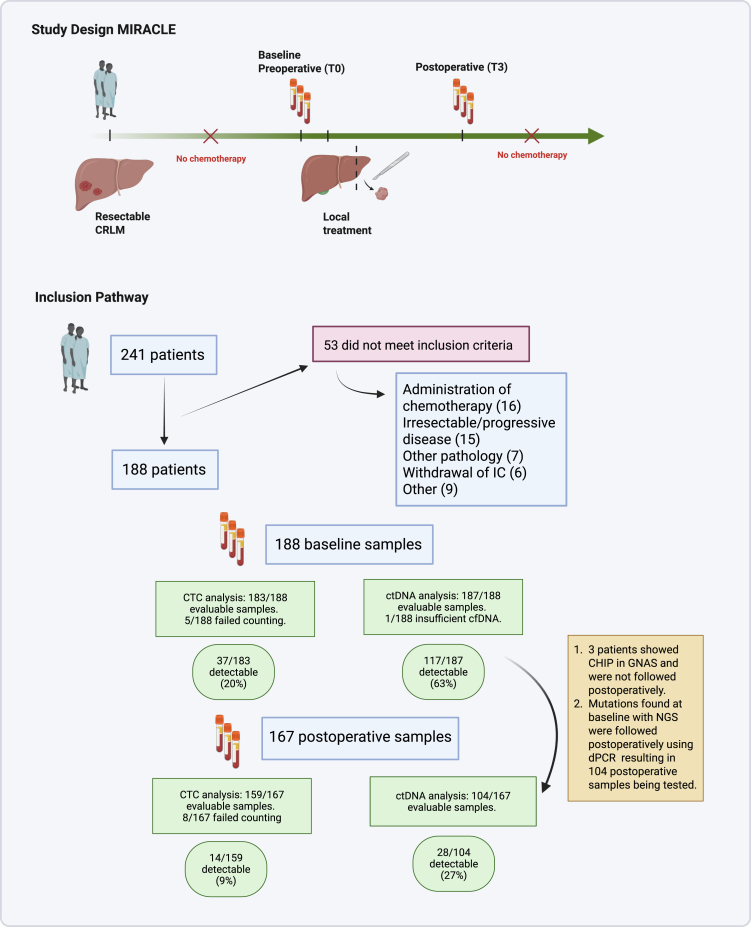
Study design and inclusion pathway. cfDNA: cell-free DNA; CHIP: Clonal haematopoiesis of indeterminate potential; CRLM: colorectal liver metastases; CTC: Circulating Tumour Cells; ctDNA: circulating tumour DNA; dPCR: Digital PCR; HGS: Next-Generation Sequencing.

The MIRACLE was a prospective, observational biomarker study in which 231 patients with resectable CRLM were to be included following the sample size calculation. Four Dutch hospitals participated in accrual: Erasmus University Medical Centre, Amphia hospital, IJsselland hospital and Albert Schweitzer hospital. Potential participants were identified through multidisciplinary team meetings and in the outpatient clinic of the surgical departments of each hospital. To be eligible for inclusion, patients had to have a history of histologically confirmed colorectal cancer and had to be currently diagnosed with resectable, isolated CRLM. The current standard of care according to the national guidelines for these patients in the Netherlands is local treatment of the CRLM without perioperative chemotherapy. Patients with extrahepatic disease, with liver-first treatments or with CRLM deemed irresectable at that point were not included. Patients participating in any other studies in which they would receive perioperative chemotherapy were not included. A total of 241 patients were prospectively enrolled between October 2015 and December 2021. Fifty-three patients were excluded for multiple reasons as illustrated in Fig. 1, leaving a total of 188 for further analysis.

### Ethics

The protocol was approved by the institutional ethics committee (MEC-2015-289) at the initiating centre (Medisch Ethische Toetsings Commissie at the Erasmus University Medical Centre, Rotterdam, The Netherlands), and at each participating site. All participants provided written informed consent in accordance with the principles of the Declaration of Helsinki.

### Procedures

Blood collection was conducted preoperatively (baseline, T0) and at 3 weeks during the postoperative outpatient visit at the surgery department (postoperative, T3), as illustrated in Fig. 1. Baseline samples were collected prior to surgery at the surgical theatre or the evening before surgery at the surgical ward. Each time 30 mL of blood was collected in Cellsave tubes. Samples from the peripheral inclusion centers were collected and transported to the research lab of Medical Oncology (Translational Cancer Genomics Lab) to be processed within 96 h for CTC enumeration and plasma isolation in parallel according to our previously established procedures.^17^^,^^18^ Cell-free DNA (cfDNA) was extracted from 4 mL plasma using either the Maxwell RSC ccfDNA Plasma kit (Promega) or the QiaAMP CNA kit (Qiagen) according to the manufacturer’s instructions. The resulting cfDNA was quantified by the Qubit 2.0 dsDNA high sensitivity assay (Thermo Fisher Scientific), and stored at −30 °C until further molecular analyses. For CTC enumeration the buffycoat of 30 mL CellSave blood was collected and supplemented to 7.5 mL with dilution buffer.

CTCs were enumerated using the FDA-approved CellSearch™ system following the manufacturer’s instructions (Menarini Silicon Biosystems, Castel Maggiore, Italy). CTC enumeration results were reviewed by 2 trained operators.^17^ CTCs were selected based on the following criteria: size ≥  4 μm, round to oval morphology, positive staining for CK8, 18 or 19, a visible DAPI positive nucleus, at least 50% overlap between nucleus and cytoplasm and negative staining for CD45. A sample was considered positive when >2 CTCs were identified in 30 mL of whole blood, as was previously described for metastatic colorectal cancer.^19^

Baseline cfDNA was evaluated using the Oncomine colon cfDNA Assay (Thermofisher) for targeted sequencing of hotspot regions from 14 colon cancer specific driver genes (Supplementary Table S1). Briefly, Next-generation sequencing (NGS) libraries were prepared from 10 ng cfDNA, sequenced on an IonTorrent S5 system to a median 20,000 × read depth coverage and analysed by Torrent Suite (Thermofisher) using default settings for Basecaller, tmap and torrent variant caller as previously described.^20^ Mutations were called when their variant allele frequency (VAF) exceeded the variant-specific limit of detection (LoD) based on its molecular coverage. Mutations detected at baseline with NGS were verified at T0 and monitored at T3 by digital PCR (dPCR) when mutation-specific assays were available. When no dPCR assay was available, Oncomine was repeated at T3, evaluating only the mutations that were detected at baseline.

dPCR was performed using the Naica system from Stilla Technologies (Villejuif, France). cfDNA was first pre-amplified using 2 μl cfDNA input together with 4 μl Taqman PreAMP Master Mix (ThermoFisher Scientific), and 2 uL 20 × diluted assay of the specific target (containing target-specific primers; Supplementary Table S1) as described previously.^20^

The dPCR reaction, consisting of Stilla Buffer A, Stilla Buffer B, Multiplex Primer probe mix and pre-amplified cfDNA with a total input volume of 27 uL, was loaded on a Sapphire chip (Stilla Technologies). PCR was performed with the following conditions: 3 min 95 °C, 45 cycles of 10 s 95 °C and 15 s at the annealing temperature (Supplementary Table S1). Sapphire chips were scanned with the Naica Prism3 reader (Stilla Technologies) using the following exposure times for the three channels: 50 ms for FAM, 250 ms for HEX and 20 ms for Cy5. Total crystal enumeration and quality control was performed by the Crystal Reader software. The extracted fluorescence values for each crystal were further analysed using the Crystal Miner software (Stilla Technologies, Villejuif, France).

dPCR experiments were repeated when less than 500 positive droplets were generated (i.e., droplets containing wildtype and/or mutant signal) or when more double positive droplets were observed than mutant droplets (overloading). The median number of signal-containing droplets in our experiments was 4585 (range: 566-23706). The VAF of each mutation in a sample was calculated by summing the single and double positive droplet signals and dividing it by the total positive droplet signal (i.e., single mutant positive, single wildtype positive and double positive droplets together). To determine the threshold for positivity for all used dPCR assays, cfDNA from plasma of at least 10 healthy blood donors (HBDs; 5 females and 5 males; median age of 36 (range: 20–76)) was analysed for each dPCR assay. The mean VAF plus one standard deviation (SD) in these 10 HBDs was used as the positivity threshold (Supplementary Table S1). To reduce the risk of false positive results due to clonal haematopoiesis, germline DNA from white blood cells were analysed on those cases where a similar VAF was observed before and after surgery in genes known to be affected by clonal haematopoiesis. This resulted in the exclusion of 3 patients with a single mutation at amino acid position 201 of GNAS.^21^

### Statistics

The primary objective of this study was to assess the potential association between postoperative ctDNA detection and recurrence of disease for patients with resectable CRLM within one year after resection, both locoregional and distant recurrence. Based on the observed mutation frequencies in primary colorectal cancer in The Cancer Genome Atlas (TCGA) at least 50% of patients were expected to have a detectable mutation in their blood covered by the utilised targeted panel (NGS, Oncomine CRC panel). Recurrence rates of patients treated with CRLM are estimated to be around 50% within the first year and 20–40% already occurs within the first 6 months postoperatively.^22^^,^^23^ We postulated we should reach a postoperative ctDNA detection rate of at least 30% and a 25% increased recurrence risk in ctDNA-positive patients within 1 year to establish clinical relevance of ctDNA in this setting. Using the Chi-squared statistic with continuity correction for dichotomous outcomes, at least 81 informative patients were required for 80% power at a 5% significance level. Considering the estimated baseline positivity of 50% and an expected drop-out rate of around 30%, we aimed to include at least 231 patients. Secondary aims were the association between recurrence within one year after local treatment and the detection of 1) baseline ctDNA, 2) baseline CTCs and 3) postoperative CTCs.

Baseline characteristics were compared using Fisher’s exact test for discrete variables and the Kruskal–Wallis test for continuous variables. Continuous variables are represented as median and IQR. Recurrence-free survival (RFS) was defined as the time from liver resection until diagnosis of locoregional or distant relapse on radiographical imaging or until last follow-up. Time-to-event analysis were computed through the Kaplan–Meier method resulting in survival curves. Log-rank test was computed for the primary aim 1-year RFS. Sensitivity Kaplan Meier analyses taking missing samples into account were conducted for ctDNA and CTCs. Univariable and multivariable Cox proportional hazard analyses were performed for 1-year RFS. Known and prespecified confounders were adjusted for. The FONG clinical risk score consists of five preoperative clinical variables: Node-positive primary, disease-free interval between the primary tumour and diagnosis of CRLM (<12 months), preoperative CEA levels (above 200 ng/mL), diameter (above 5 cm) and number of the CRLM (above 1 lesion).^24^ When patients have one or two out of five risk factors, they are scored low, as opposed to three to five risk factors scoring high. These results were reported as hazard ratios (HR) with 95% confidence intervals (CI). A two-sided p value of less than 0.05 was considered statistically significant. This analysis was based on the analysable population and, therefore, no imputation was carried out. The proportional hazards assumption was examined in all the included Cox models. All statistical analyses were performed using R software version 4.2.3 (R project for Statistical Computing).

### Role of funding source

The funder of the study (Dutch Cancer Society, EMCR 2014-6340) had no role in study design, data collection, data analysis, data interpretation, or writing of the report.

## Results

Clinicopathologic characteristics of patients included in the analysis are shown in Table 1. The median patient age was 67 years [IQR 61–74 years] and 69% were male. Sixty-one percent of patients had a node positive primary, 37% had synchronous disease, and 47% had more than 1 liver metastasis. Per our inclusion criteria, no patients received upfront or postoperative chemotherapy. An R0 liver resection was achieved in 170 (91%) patients. Median follow-up was 37 months [IQR 26–53 months] and a total of 106 (57%) patients experienced recurrence within one year. Collection time points of samples within the cohort are shown in Fig. 1. Baseline blood samples were collected prior to surgery at the surgical theatre or the evening before surgery at the surgical ward, and the postoperative blood sample was collected with a median of 18 days after surgery [IQR 15–22 days].

**Table 1:** Baseline characteristics.

	Overall	ctDNA		p-value	CTC		p-value
		Baseline+	Baseline−		Baseline+	Baseline−	
N	188	117	70		37	146	
**Sex, N (%)**							
Female	58 (30.9)	36 (30.8)	21 (30.0)	0.91	12 (32.4)	45 (31.0)	0.87
Male	130 (69.1)	81 (69.2)	49 (70.0)		25 (67.6)	101 (69.0)	
Age at resection CRLM (median [IQR])	67.0 [61.0, 74.0]	66.0 [60.0, 72.0]	67.0 [61.0, 75.0]	0.38	67.0 [61.0, 74.0]	66.0 [61.0, 73.8]	0.74
**ASA class, N (%)**							
1–2	132 (70.2)	80 (68.4)	51 (72.9)	0.52	25 (67.6)	103 (70.5)	0.72
3–4	56 (29.8)	37 (31.6)	19 (27.1)		12 (32.4)	43 (29.5)	
**Location primary, N (%)**							
Left-sided	79 (42.0)	45 (38.5)	33 (47.1)	0.49	18 (48.6)	62 (42.5)	0.54
Rectum	64 (34.0)	43 (36.8)	21 (30.0)		13 (35.1)	48 (32.9)	
Right-sided	45 (24.0)	29 (24.7)	16 (22.9)		6 (16.3)	36 (24.7)	
**T stage primary, N (%)**							
1–2	33 (17.6)	23 (19.7)	10 (14.3)	0.35	5 (13.5)	27 (18.5)	0.45
3–4	152 (80.8)	92 (78.6)	59 (84.3)		32 (86.5)	116 (79.5)	
Unknown	3 (1.6)	2 (1.7)	1 (1.4)		0 (0.0)	3 (2.0)	
**Lymphnodes primary,N (%)**							
N0	71 (37.8)	43 (36.8)	27 (38.6)	0.81	10 (27.0)	58 (39.7)	0.13
N+	114 (60.6)	72 (61.5)	42 (60.0)		27 (73.0)	85 (58.3)	
Unknown	3 (1.6)	2 (1.7)	1 (1.4)		0 (0.0)	3 (2.0)	
**Metachronous/synchronous, N (%)**							
Metachronous	119 (63.3)	75 (64.1)	44 (62.9)	0.86	26 (70.3)	91 (62.3)	0.37
Synchronous	69 (36.7)	42 (35.9)	26 (37.1)		11 (29.7)	55 (37.7)	
**Disease-free interval primary—CRLM, N (%)**							
≤1 Year	126 (67.0)	77 (65.8)	48 (68.6)	0.70	25 (67.6)	97 (66.4)	0.90
>1 Year	62 (33.0)	40 (34.2)	22 (31.4)		12 (32.4)	49 (33.6)	
**Number CRLM, N (%)**							
1	97 (51.6)	55 (47.0)	41 (58.6)	0.13	14 (37.8)	81 (55.5)	0.055
>1	91 (48.4)	62 (53.0)	29 (41.4)		23 (62.2)	65 (44.5)	
**Diameter largest CRLM (cm), N (%)**							
≤5	173 (92.0)	104 (88.9)	68 (97.1)	0.061	30 (81.1)	138 (94.5)	0.0042^a^
>5	14 (7.5)	12 (10.3)	2 (2.9)		7 (18.9)	7 (4.8)	
Unknown	1 (0.5)	1 (0.8)	0 (0.0)		0 (0.0)	1 (0.7)	
**Elevated CEA before resection CRLM, N (%)**							
<5	48 (25.5)	29 (24.8)	19 (27.1)	0.94	11 (29.7)	35 (24.0)	0.64
>5	109 (58.0)	66 (56.4)	42 (60.0)		22 (59.5)	85 (58.2)	
Unknown	31 (16.5)	22 (18.8)	9 (12.9)		4 (10.8)	26 (17.8)	
**Kras status (tissue), N (%)**							
mt	33 (17.6)	26 (22.2)	6 (8.6)	0.0050	5 (13.5)	27 (18.5)	0.53
wt	37 (19.7)	18 (15.4)	19 (27.1)		8 (21.6)	29 (19.9)	
Unknown	118 (62.8)	73 (62.4)	45 (64.3)		24 (64.9)	90 (61.6)	
**TP53 status (tissue), N (%)**							
mt	25 (13.3)	16 (13.7)	9 (12.9)	0.86	6 (16.2)	18 (12.3)	0.72
wt	15 (8.0)	10 (8.5)	5 (7.1)		3 (8.1)	12 (8.2)	
Unknown	148 (78.7)	91 (77.8)	56 (80.)		28 (75.7)	116 (79.5)	
**MSI status, N (%)**							
MSI	3 (1.6)	2 (1.7)	1 (1.4)	0.84	0 (0.0)	3 (2.0)	0.38
MSS	103 (54.8)	62 (53.0)	40 (57.2)		21 (56.8)	80 (54.8)	
Unknown	82 (43.6)	53 (45.3)	29 (41.4)		16 (43.2)	63 (43.2)	
**Riskgroup fong, N (%)**							
High	50 (26.6)	37 (31.6)	13 (18.6)	0.049^a^	15 (40.5)	34 (23.3)	0.041^a^
Low	135 (71.8)	78 (66.7)	56 (80.0)		22 (59.5)	109 (74.7)	
Unknown	3 (1.6)	2 (1.7)	1 (1.4)		0 (0.0)	3 (2.0)	
**Resection margin CRLM, N (%)**							
R0	170 (90.4)	105 (89.7)	64 (91.4)	0.19	32 (86.5)	135 (92.5)	0.30
R1	11 (5.9)	9 (7.7)	2 (2.9)		3 (8.1)	6 (4.1)	
Unknown	7 (3.7)	3 (2.6)	4 (5.7)		2 (5.4)	5 (3.4)	

One sample had insufficient cfDNA for analyses at baseline. CTC enumeration failed in five baseline samples.

CRLM: colorectal liver metastases; ctDNA: circulating tumour DNA; CTC: circulating tumour cells; IQR: Interquartile range; MSI: Microsatellite Instability.

a Significant result.

**Fig. 2:**
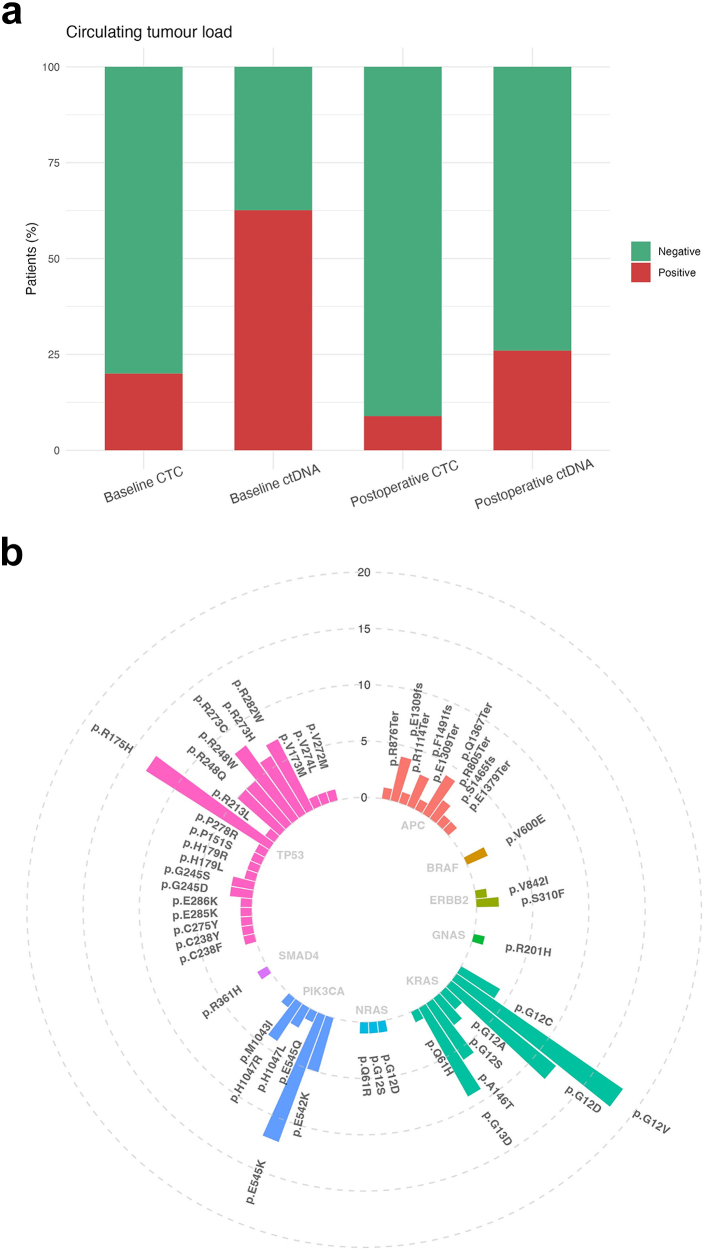
Panel A. Detection rates of CTC and ctDNA. Baseline CTC: 37/183 (20%); Baseline ctDNA: 117/187 detectable (63%); Postoperative CTC: 14/159 detectable (9%); Postoperative ctDNA: 28/104 detectable (27%). CTC: Circulating Tumour Cells; ctDNA: circulating tumour DNA. Panel B. The number of detected mutations (circles) at baseline through next-generation sequencing of blood samples.

**Fig. 3:**
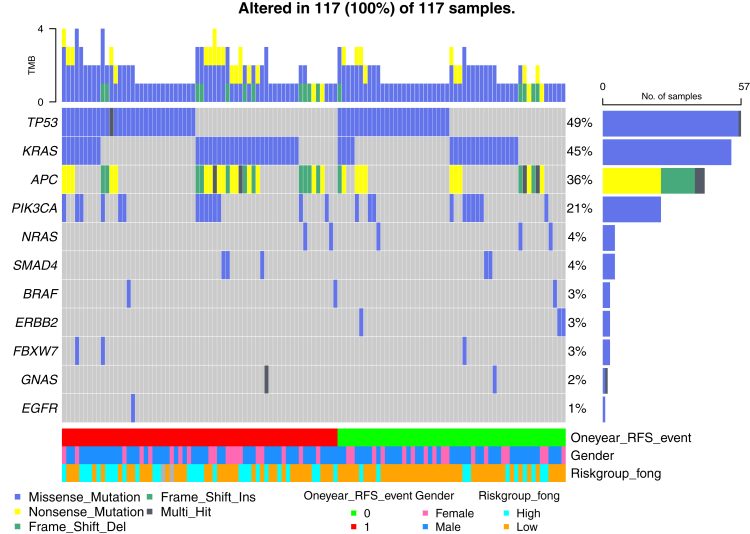
Oncoplot depicting the identified mutations by next-generation sequencing of preoperative blood samples. Oneyear_RFS_event: recurrence within one year after local treatment.

Out of 188 included patients for analysis, one sample had insufficient cfDNA for analyses at baseline and CTC enumeration failed in five baseline samples. A total of 117 out of 187 patients (63%) with evaluable samples showed detectable ctDNA at baseline (T0) by NGS analysis (Fig. 2a) and NGS-identified mutations were validated by dedicated dPCR analyses in all but one patient, showing a strong correlation between the obtained VAFs by the Oncomine panel and dPCR analyses (Spearman’s Rho = 0.82). Details on the mutations found at baseline in this patient group are illustrated in Fig. 2b and 3. Patients with a detected mutation in KRAS at baseline had a shorter 1-year RFS compared to all other patients in the cohort (1-year RFS 37% [95% CI 26%–53%] vs. 55% [95% CI 47%–64%], log-rank p = 0.028), as shown in Supplementary Fig. S1. This difference in RFS became non-significant when restricting the analysis to only patients who showed detectable ctDNA at baseline (1-year RFS 37% [95% CI 26%–53%] vs. 51% [95% CI 40%–65%], log-rank p = 0.13, Supplementary Fig. S1). From the 117 verified ctDNA-positive patients at baseline, 27% remained ctDNA positive postoperative (T3, Fig. 2a). Remaining mutations were found in TP53 (50% of postoperative ctDNA-positive cases), KRAS (32%), PIK3CA (11%), and APC (7%). A median 18-fold reduction was found in VAF compared to the baseline value (Supplementary Fig. S2). The median VAF of baseline positive patients was 5.96 [IQR 2.38–15.31] at baseline and 0.00 [IQR 0.00–0.12] at timepoint T3. The subgroup of patients who were positive at T3 had a median VAF of 0.50 [IQR 0.22–0.97].

**Fig. 4:**
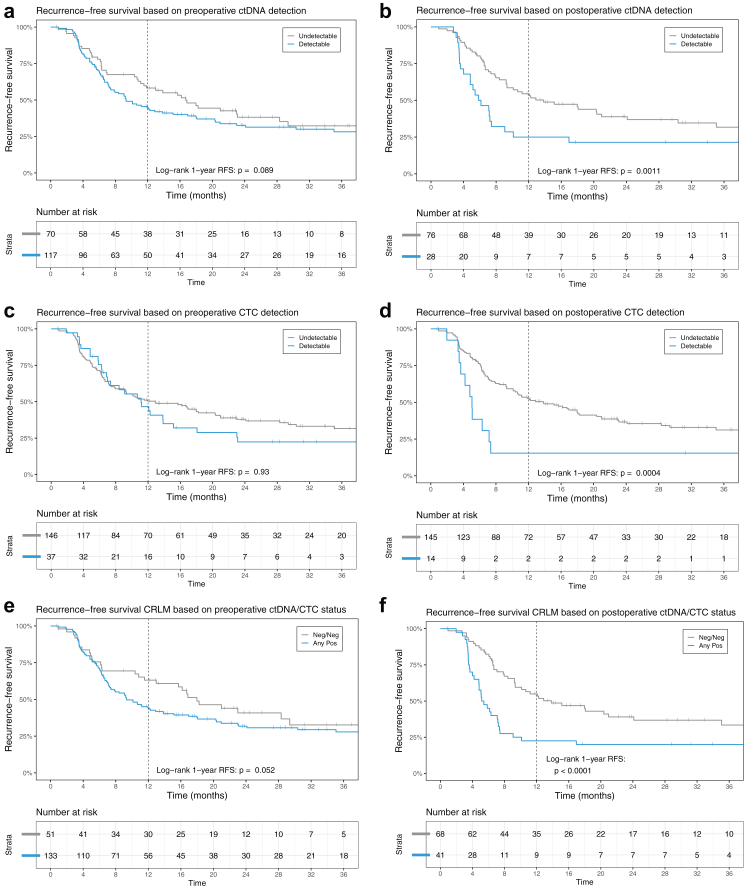
Panel A. Recurrence-free survival through Kaplan–Meier method: preoperative ctDNA (mutations). Panel B. Recurrence-free survival through Kaplan–Meier method: postoperative ctDNA (mutations). Panel C. Recurrence-free survival through Kaplan–Meier method: preoperative CTC enumeration. Panel D. Recurrence-free survival through Kaplan–Meier method: postoperative CTC enumeration. Panel E. Recurrence-free survival through Kaplan–Meier method: combined preoperative ctDNA/CTC status. Panel F. Recurrence-free survival through Kaplan–Meier method: combined preoperative ctDNA/CTC status.

Patients with baseline detectable ctDNA had a comparable RFS to patients without ctDNA detected (1-year RFS: 45% [95% CI 36%–55%] vs. 58% [95% CI 48%–71%], log-rank test p = 0.089, Fig. 4a). Patients with postoperative detectable ctDNA at three weeks postoperatively (T3) had a significantly shorter RFS (1-year RFS: 25% [95% CI 13%–47%] vs. 54% [95% CI 44%–67%], log-rank test p = 0.0011, Fig. 4b). In other words, a total of 75% of patients with detectable ctDNA showed recurrence within one year after surgery, as opposed to 46% of patients without detectable ctDNA after surgery. Sensitivity Kaplan Meier analyses taking missing samples/analyses into account were conducted (Supplementary Figs. S3 and S4) and showed no differences in the primary endpoint 1-year RFS. No evident differences were found in the intrahepatic and extrahepatic recurrence patterns of patients with detectable ctDNA at baseline or postoperatively.

A total of 37 out of 183 patients (20%) showed detectable CTCs at baseline (T0) and postoperative detectable CTCs were found in 14 out of 158 (9%) evaluable samples at T3 (Fig. 2a). The median CTC count at baseline was 0 [IQR 0–2] and postoperatively 0 [IQR 0–0]. The subgroup of patients who were positive at baseline had a median CTC count of 5 [IQR 3–10] and the group of patients who tested positive postoperatively (T3) had a median CTC count of 5 [IQR 3–8].

Patients with and without detectable CTCs at baseline had a similar RFS (1-year RFS: 50% [95% CI 43%–60%] vs. 47% [95% CI 33%–66%], log-rank test p = 0.93, Fig. 4c). Patients with postoperatively detectable CTCs had a significantly shorter RFS (1-year RFS: 15% [95% CI 4%–55%] vs. 53% [95% CI 45%–62%], log-rank test p = 0.0004, Fig. 4d) than patients without detectable CTCs. In fact, out of 14 patients with detectable CTCs postoperatively, 85% showed recurrence within one year after surgery, as opposed to 47% of patients without detectable CTCs after surgery. Sensitivity Kaplan Meier analyses taking missing samples/analyses into account were conducted (Supplementary Fig. S5) and showed no differences in the primary endpoint 1-year RFS. No evident differences were found in the intrahepatic and extrahepatic recurrence patterns of patients with detectable CTCs at baseline or postoperatively.

**Fig. 5:**
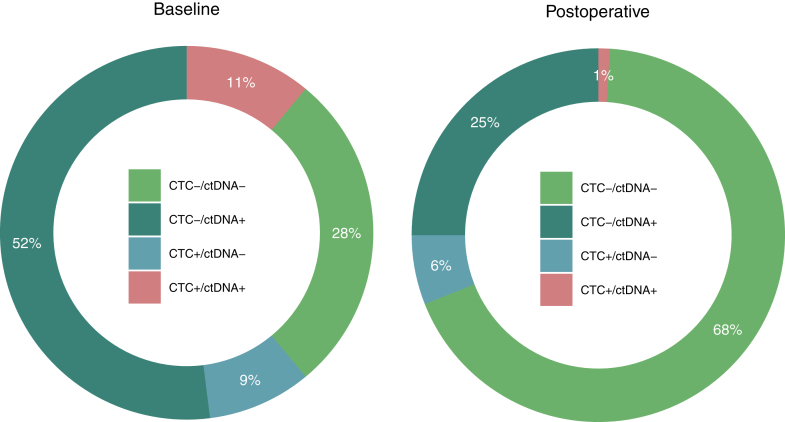
Biomarkers combined in groups at baseline and postoperatively. Baseline N = 182; postoperative N = 98. CTC: Circulating Tumour Cells; ctDNA: circulating tumour DNA.

For a large portion of participants both CTCs and ctDNA were analysed before surgery (n = 184) and after surgery (n = 98). Combination of the biomarkers is visually represented in Fig. 5. Over half of patients had CTC−/ctDNA+ (52%) at baseline. The largest group postoperatively was CTC−/ctDNA-entailing 68% of patients. Patients with any detectable circulating tumour load (CTCs and/or ctDNA) at baseline had a shorter 1-year RFS, though not statistically significant, as opposed to patients who had no circulating tumour load at all (1-year RFS: 44% [95% CI 36%–54%] vs. 63% [95% CI 51%–78%], log-rank test p = 0.052, Fig. 4e). Postoperative detection of any circulating tumour load lead to a significantly shorter 1-year RFS of 23% [95% CI 13%–40%] for patients with any detectable circulating tumour load (CTCs and/or ctDNA) compared to 55% [95% CI 44%–68%] for patients with no detectable circulating tumour load (log-rank test p < 0.0001, Fig. 4f).

In this patient cohort, postoperative ctDNA and/or CTCs, the combined Fong clinical risk score^24^ and primary tumour location was found to be significantly associated with 1-year RFS in univariable analysis (Table 2). Correcting for prespecified clinical risk factors (Fong clinical risk score and location primary tumour), multivariable analysis revealed a significantly shorter 1-year RFS in patients with postoperative detectable CTCs (HR 2.98; 95% CI 1.56–5.71; p = 0.0010) and ctDNA (HR 2.35; 95% CI 1.34–4.11; p = 0.0028) compared to patients with undetectable CTC and ctDNA, respectively. Similarly, any postoperative circulating tumour load (ctDNA and/or CTCs) was also a predictor for 1-year recurrence (HR 2.77; 95% CI 1.64–4.66; p = 0.0001).

**Table 2:** Univariable and multivariable Cox proportional hazard model for 1-year recurrence-free survival per biomarker.

Variable	Univariable		Multivariable ctDNA (N = 102)		Multivariable CTC (N = 156)		Multivariable ctDNA/CTC (N = 107)	
	HR (95% CI)	p-value	HR (95% CI)	p-value	HR (95% CI)	p-value	HR (95% CI)	p-value
Age at resection (continuous)	0.99 (0.97–1.01)	0.47						
Fong risk score	2.15 (1.41–3.29)	0.0004^a^	2.73 (1.57–4.72)	0.0003^a^	2.72 (1.70–4.35)	<0.0001^a^	2.29 (1.35–3.88)	0.0022^a^
Inclusion site								
Albert Schweitzer (ref)								
Amphia	1.72 (0.76–3.79)	0.18						
ErasmusMC	1.19 (0.55–2.54)	0.66						
IJsselland	1.95 (0.86–4.43)	0.11						
Location primary tumour								
Left-sided (ref)								
Right-sided	1.12 (0.69–1.81)	0.66	1.43 (0.73–2.79)	0.29	1.16 (0.68–1.99)	0.58	1.56 (0.83–2.93)	0.17
Rectum	0.54 (0.33–0.91)	0.020^a^	0.70 (0.37–1.32)	0.27	0.47 (0.26–0.84)	0.011^a^	0.70 (0.38–1.30)	0.26
Resection margin								
R1	1.50 (0.70–3.25)	0.30						
KRAS (tissue)	1.06 (0.61–1.84)	0.83						
BRAF (tissue)	0.77 (0.11–5.61)	0.80						
MSI	0.52 (0.07–3.72)	0.51						
Baseline								
Preoperative ctDNA	1.47 (0.94–2.29)	0.091						
Preoperative CTC	1.02 (0.62–1.70)	0.94						
Preoperative circulating tumour load (ctDNA or CTC)	1.24 (0.79–1.94)	0.36						
Postoperative								
Postoperative ctDNA	2.41 (1.40–4.18)	0.0016^a^	2.35 (1.34–4.11)	0.0028^a^				
Postoperative CTC	3.01 (1.58–5.73)	0.0008^a^			2.98 (1.56–5.71)	0.0010^a^		
Postoperative circulating tumour load (ctDNA or CTC)	2.78 (1.67–4.61)	<0.0001^a^					2.77 (1.64–4.66)	0.0001^a^

ctDNA: circulating tumour DNA; CTC: circulating tumour cells; MSI: Microsatellite Instability.

a Significant result.

## Discussion

In this cohort of resectable CRLM patients without any perioperative chemotherapy, detection of postoperative CTCs and ctDNA alone or combined were independent risk factors for a shorter RFS. To date, this study included the largest cohort of CRLM patients with both CTC and ctDNA analyses before and after resection. Additionally, this is the first study to investigate these promising biomarkers in chemonaive, resectable CRLM patients resulting in a homogenously treated cohort and a circulating tumour load unaffected by any (neo)adjuvant chemotherapy treatment.

The mere presence of circulating tumour load before surgery showed no clear prognostic value in this cohort. Similarly, Newhook et al. showed no significant association with preoperative ctDNA detection and RFS or OS in a CRLM cohort of 48 patients.^16^ In contrast, Kobayashi et al. did find a significantly shorter RFS in patients with preoperative ctDNA; but this did not translate into a significantly shorter OS.^25^ Differences in findings may possibly be explained by heterogenous chemotherapy schemes, varied analysis methods and detection thresholds. Even though the presence of ctDNA itself was not prognostic for RFS at baseline in this cohort, this does not necessarily imply that ctDNA at baseline cannot be of prognostic value. There may be potential significance in the levels of the detected mutations at baseline, meaning patients with high detectable circulating tumour load may in fact have worse outcome. For instance, Pairawan et al. found that the highest quartile of VAF was associated with worse OS in patients with metastatic disease^26^ and Elez et al. showed that a VAF under 5.8% in patients with metastatic colorectal cancer statistically improved OS.^27^ Postoperative results in the MIRACLE cohort are in line with previously conducted studies in smaller cohorts and with a wide range in different treatment strategies including (neo)adjuvant chemotherapy.^14^, ^15^, ^16^^,^^28^ The GALAXY trial, in which a tumour-informed, personalised ctDNA assay was designed, reported similar postoperative ctDNA detection rates as in this current cohort (32% vs. 27%, respectively).^28^ In terms of recurrence risk, Tie et al. showed comparable results in a cohort of 54 patients for whom detectable postoperative ctDNA resulted in a significantly lower RFS (HR 6.3; p < 0.001).^14^ Similarly, Ogaard et al. established that ctDNA status was a stronger predictor of recurrence than standard clinical risk factors and that patients with postoperative ctDNA experiences a significantly shorter RFS (HR 4.5; p < 0.001).^15^

CTCs and ctDNA compose two different approaches, each with advantages and limitations. CTCs may be relevant for early detection due to their metastatic potential, however comprehensive investigation is hindered by the low numbers of CTCs present in patients with CRC.^29^ Additionally, the FDA-approved CellSearch® assay targets only EpCAM-positive CTCs. Lastly, due to its complexity, trained observers are needed for CTC counting, potentially hindering its implementation in standard practice. ctDNA tends to be less complex to isolate and exhibits a higher positivity rate compared to CTCs, rendering ctDNA analyses more straightforward for implementation into clinical practice. ctDNA has the advantage of capturing tumour heterogeneity and provides a comprehensive genetic map, as opposed to CTC enumeration. Nonetheless, mutation detection in cfDNA may include non-tumour-specific, age-dependent alterations in common driver genes, known as clonal haematopoiesis, and there is still a lack in specific analysis standards across clinics and laboratories. In our cohort, we identified mutations derived from clonal haematopoiesis in 3 patients. Lastly, detecting at least one biomarker (CTCs or ctDNA) postoperatively was linked to a higher recurrence risk within the first year, highlighting the potential benefit of dual biomarker analysis in detecting all patients with a high risk for recurrence. Even though dual biomarker testing may detect more patients with circulating tumour load, and thus inferior RFS, than a singular marker, it also leads to higher costs and longer turnaround time in the clinic. Additionally, testing for both biomarkers may not always be possible due to lack of accessibility to a specialised lab and experienced personnel. On the other hand, a tumour-informed panel, though higher in sensitivity, is even more complex and would result in even higher costs and longer turnaround times. Approaches like dual biomarker assessment may offer an intermediate solution, maximising the advantages while mitigating some drawbacks, and could be more feasible for initial implementation in clinical practice. In the future, tumour-informed strategies are expected to become more accessible.

The use of systemic therapies for initially resectable CRLM continues to be a topic of debate. A recent study showed that perioperative FOLFOX does not improve RFS in patients with resectable CRLM as opposed to resection and adjuvant chemotherapy.^30^ Additionally, the EPOC randomised controlled trial investigating surgery alone vs. perioperative FOLFOX showed only a trend towards improved RFS for the perioperative chemotherapy group, though no significant benefit in OS.^6^ Similarly, other trials like the JCOG0603, a phase III randomised controlled trial, and the FFCD ACHBTH AURC 9002 Trial, were also unable to show an OS benefit of adjuvant chemotherapy.^31^^,^^32^ We hypothesise that a subset of patients with resectable CRLM may derive benefit from perioperative chemotherapy, as is common practice in large parts of the world. However, analysis of the outcomes from the randomised controlled trials indicates that this benefit is not uniformly observed across all patients with resectable CRLM. According to international guidelines, perioperative systemic therapy is considered or even recommended, especially in patients with a high risk for recurrence.^33^^,^^34^ Current risk score systems like FONG for patients with CRLM include solely preoperative clinical risk factors.^24^ The MIRACLE cohort shows that novel, real-time biomarkers reflecting microscopic disease, like ctDNA, can stratify patients into risk for recurrence. Just like in the primary setting, ctDNA may have the potential to guide clinicians and patients in perioperative therapy strategy decision-making. A recent subgroup analysis from the GALAXY trial by Katoaka et al. showed the potential benefit of adjuvant chemotherapy in RFS in a small cohort of patients with ctDNA detected postoperatively.^28^ Together, results from the GALAXY and MIRACLE strongly support the initiation of a randomised trial to determine the clinical benefit of ctDNA-guided adjuvant chemotherapy in this patient population. This could contribute to more personalised treatment strategies and may translate into an overall survival benefit.

The following limitations to this study should be considered. Analyses of ctDNA were conducted using a single colon-specific targeted panel, without prior knowledge of the genomic profile of the tumour tissue. This approach limited the number of evaluable patients to those carrying a detectable mutation in one of the covered genes at baseline (63% of patients), but also has multiple advantages such as shorter turnaround time and no need for patient-specific assays, design of which is complex, time-consuming and expensive. The cause for undetectable ctDNA at baseline can be twofold: 1) the utilised panel may have had insufficient coverage of the mutational landscape of the baseline-negative patients in our cohort and/or 2) certain patients may have had a very low level of ctDNA, not reaching the limit of detection. Therefore, a tumour-informed approach, though time-consuming and costly, will lead to higher sensitivity and, thus, an increased ctDNA detection rate. This was shown by Tie et al., reaching a preoperative ctDNA detection rate of 85% before any treatment (46 of 54 patients) in patients with resectable CRLM.^14^ Similarly, Kataoka et al. recently showed in a subgroup analysis from the GALAXY including only CRLM patients, that using a tumour-informed approach, 98% of patients was ctDNA positive before surgery.^28^ However, the postsurgical ctDNA detection rates were with 32% comparable to the current cohort (27% in the MIRACLE). Based on this, there is support that the postoperative results in the MIRACLE cohort provide clinical relevance of ctDNA-based MRD detection in initially resectable CRLM patients without any bias caused by perioperative systemic treatment. In addition, recent studies investigating tumour-agnostic approaches have shown promising results in terms of specificity and sensitivity, using for example colorectal cancer-specific DNA methylation markers.^15^^,^^35^^,^^36^ A recent study in patients with CRC utilised the multiomic NGS platform Guardant Reveal, which detects both tumour-derived genomic alterations and an epigenomic (methylation) signature. This study revealed increased detection rates by identifying some patients by having methylation patterns only.^36^ Novel advances such as ctDNA methylation and fragmentomics hold promise in improving ctDNA detection sensitivity and are more universal markers compared to using genetic alterations alone. For instance, Parikh et al. found that plasma-only postoperative MRD detection integrating epigenomic and genomic alterations demonstrated favorable sensitivity and specificity for recurrence, comparable to tumour-informed approaches.^35^ Lastly, the long-term results on patients with postoperative CTCs should be interpreted with due consideration given the low availability of CTCs in patients with (metastasised) colorectal cancer.

This is the first study conducted in patients with resectable CRLM without (neo)adjuvant chemotherapy, which demonstrates the impact of detectable circulating tumour load after surgery on RFS. Postoperative ctDNA and CTC detection are strong predictors for a shorter RFS after local treatment. These results pave the way for further investigations in optimising patient identification for adjuvant chemotherapy strategies.

## Contributors

Conceptualisation: CV, SS, JWMM, HMWV, SMW and DJG.

Data curation: LW, CV, DJG, SMW and SM.

Formal analysis: LW, SMW and SM.

Funding acquisition: CV, SS, JWMM and HMWV.

Investigation: LW, MPHMJ, JK, YMM, KV, VdW, CMB, MV, EJTB, PDG, MV, SS, HMWV, JWMM, DJG, SMW, CV, NA, JW, AMR, PD, JvdH, BG, DJH, PMHN, EPvdS, JH, LA, PAJM, MKB, EMJ, KTI, NV, RvM, PA and ED.

Methodology: CV, SS, JWMM, HMWV, SMW and DJG.

Project administration: LW, CV and SW.

Resources: LW, MPHMJ, JK, YMM, KV, VdW, CMB, MV, EJTB, PDG, MV, SS, HMWV, JWMM, DJG, SMW, CV, NA, JW, AMR, PD, JvdH, BG, DJH, PMHN, EPvdS, JH, LA, PAJM, MKB, EMJ, KTI, NV, RvM, PA and ED.

Software: LW, SMW and SM.

Supervision: CV, JWM, SMW and DJG.

Validation: CV, SS, JWMM, HMWV, SMW and DJG.

Visualisation: LW, CV and SMW.

Writing—original draft: LW, CV and SMW.

Writing—review & editing: LW, MPHMJ, JK, YMM, KV, VdW, CMB, MV, EJTB, PDG, MV, SS, HMWV, JWMM, DJG, SMW, CV, NA, JW, AMR, PD, JvdH, BG, DJH, PMHN, EPvdS, JH, LA, PAJM, MKB, EMJ, KTI, NV, RvM, PA and ED.

The full dataset was accessible to SMW, SM, and LW. The data was verified by YMM and KV (a subset of the clinical variables) and by SMW and LW (the full dataset with all variables, clinical and lab-related).

All authors read and approved the final version of the manuscript, and had final responsibility for the decision to submit for publication.

## Data sharing statement

Data collected for the study, including de-identified individual participant data can be made available to other researchers on reasonable request. Data inquiries should be directed to the corresponding author.

## Declaration of interests

We declare no competing interests.

## Appendix A Supplementary data

Supplementary Figs. S1–S5 and Table S1

Supplementary_MIRACLE_studyteam
