# Supplementary material for: Circulating tumour cells & circulating tumour DNA in patients with resectable colorectal liver metastases (MIRACLE): a prospective, observational biomarker study
Source: eClinicalMedicine. 2025 Aug 12;87:103406. doi: 10.1016/j.eclinm.2025.103406 (PMC12361997; doi:10.1016/j.eclinm.2025.103406)
Supplement: Supplementary Figs. S1–S5 and Table S1 [file mmc1.docx]

**Suppl Table 1.** HBD results per dPCR assay

| **Mutation** | **Assay name** | **assay number/primer sequences** | **Ta (°C)** | **Cut off based on HBDs*** |
| --- | --- | --- | --- | --- |
| TP53p.R273C | TP53 p.R273C | MT: FAM AGGCACAAACACACA \| WT: HEX AGGCACAAACACGCAC \| FW CTCTGTGCGCCGGTCTCT \| RV: TGGGACGGAACAGCTTTGA | 58 | 0.21 |
| TP53p.R282W | TP53 p.R282W | ThermoFisher Scientific: TP53_10704 | 58 | 0.20 |
| KRASp.G12C | KRAS_G12C_76bp | ThermoFisher Scientific: AN9HJKW | 58 | 0.02 |
| KRASp.G12V | KRAS p.G12V | ThermoFisher Scientific:KRAS_520 | 58 | 0.02 |
| KRASp.G12D | KRAS p.G12D | ThermoFisher Scientific:KRAS_521 | 58 | 0.15 |
| PIK3CAp.H1047R | PIK3CA p.H1047R | ThermoFisher Scientific:PIK3CA_775 | 58 | 0.46 |
| PIK3CAp.E542K | PIK3CA p.E542K | ThermoFisher Scientific:PIK3CA_760 | 58 | 0.15 |
| PIK3CAp.E545K | PIK3CA p.E545K | ThermoFisher Scientific:PIK3CA_763 | 58 | 0.09 |
| KRASp.A146T | KRAS p.A146T | MT: FAM CTGTCTTGTCTTTGTTGAT \| WT: HEX TGTCTTGTCTTTGCTGAT \| FW: CAGAAAACAGATCTGTATTTATTTCAGTGT \| RV: GGACTTAGCAAGAAGTTATGGAATTCC | 58 | 0.06 |
| KRASp.G12S | KRAS p.G12S | ThermoFisher Scientific:KRAS_517 | 58 | 0.13 |
| KRASp.G13D | KRAS p.G13D | ThermoFisher Scientific:KRAS_532 | 58 | 0.22 |
| TP53p.G245S | TP53 p.G245S | ThermoFisher Scientific: ANRWFAR | 58 | 0.11 |
| TP53p.R175H | TP53 p.R175H | ThermoFisher Scientific:TP53_R175H | 58 | 0.20 |
| TP53p.R248Q | TP53p.R248Q | ThermoFisher Scientific:TP53_10662 | 58 | 0.18 |
| TP53p.R248W | TP53p.R248W | MT: FAM AGGATGGGCCTCCGGTT \| WT: HEX ATGGGCCTCCAGTT \| FW: GGAGTCTTCCAGTGTGATGATGGT \| RV: AACTACATGTGTAACAGTTCCTGCAT | 58 | 0.08 |
| TP53p.R273H | TP53p.R273H | ThermoFisher Scientific:TP53_10660 | 58 | 0.15 |
| NRASp.Q61R | NRASp.Q61R | ThermoFisher Scientific:NRAS_584 | 58 | 0.13 |
| TP53p.C275Y | TP53p.C275Y | ThermoFisher Scientific: HI15TU | 58 | 0.37 |
| KRASmultiplexassay | ddPCR KRAS G12/G13 Screening Kit | Bio-Rad: 1863506 | 52 | 0.27 |

HBD: Healthy Blood Donor; MT: Mutant probe; WT: Wildtype probe; FW: Forward primer; RV: Reverse primer; Ta: Annealing’s temperature.

**Suppl Figure 1.** Recurrence-free survival (RFS) through Kaplan-Meier method: preoperative circulating tumour DNA (ctDNA) detection through KRAS mutation**. Panel A**: complete cohort, comparing RFS of patients with a confirmed KRAS mutation and the RFS of all other patients included in the cohort. **Panel B**: subgroup of patients with detectable mutations at baseline through ctDNA analysis, comparing RFS of patients with a confirmed KRAS mutation and the RFS of patients with a confirmed wildtype.


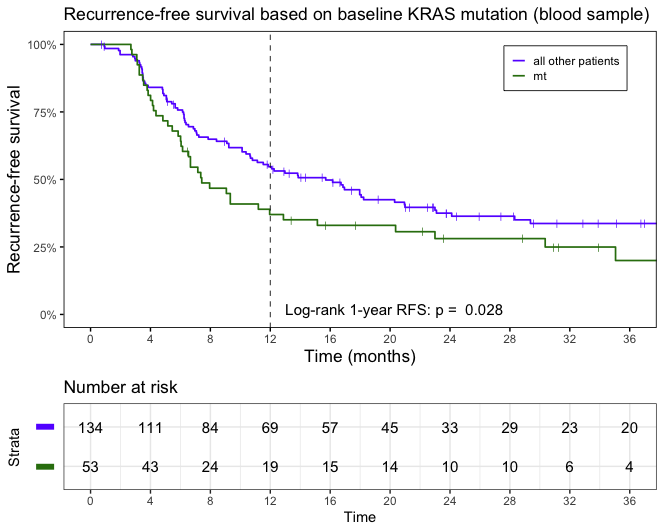

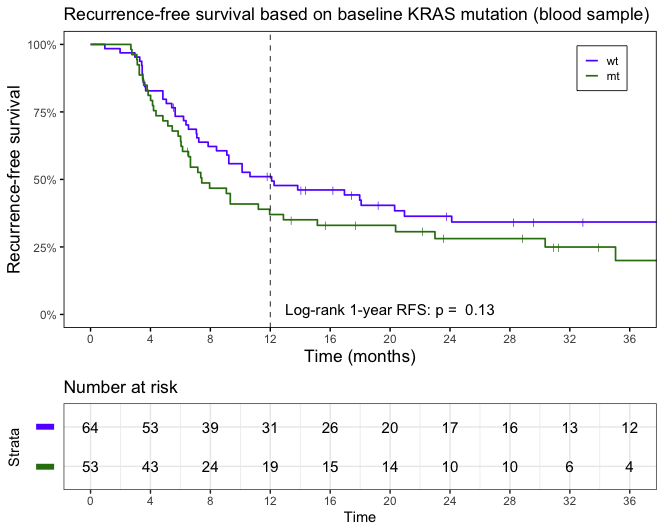


**Suppl Figure 2.** The observed variant allele frequencies (VAFs) over time. Remaining mutations postoperatively were found in TP53 (50% of postoperative ctDNA-positive cases), KRAS (32%), PIK3CA (11%), and APC (7%). A median 18-fold reduction was found in VAF compared to the baseline value.


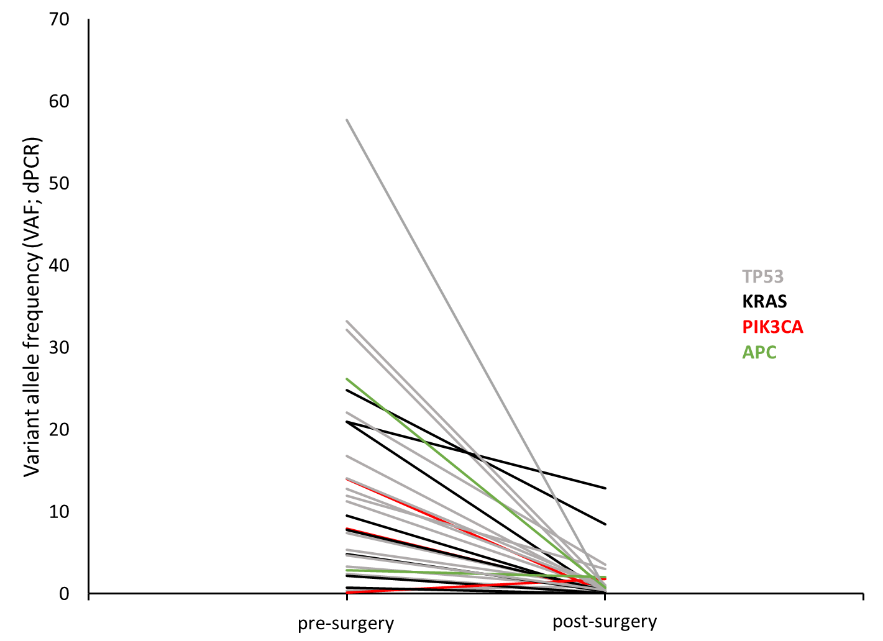


**Suppl Figure 3.** Sensitivity analysis for patients without a matched post-operative sample available. No difference in recurrence-free survival (RFS) was observed between patients with and without a post-operative blood sample.


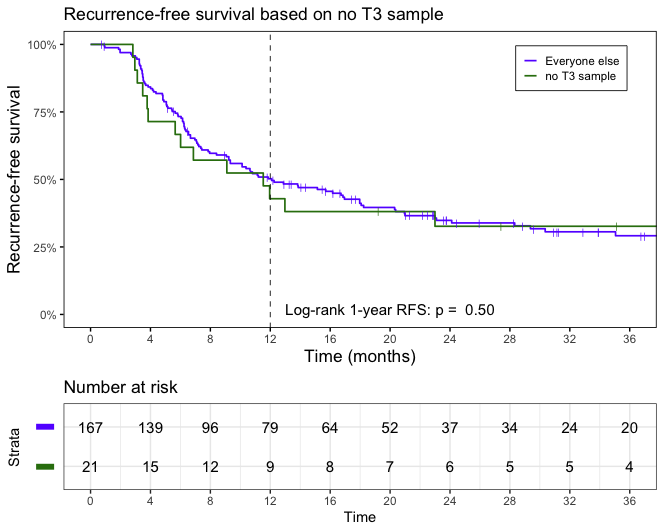


**Suppl Figure 4.** Sensitivity analysis for patients with no postoperative sample analysis for circulating tumour DNA (ctDNA) due to either the unavailability of a postoperative sample or the fact that these patients had no mutation at baseline (and thus no postoperative analysis could be done).


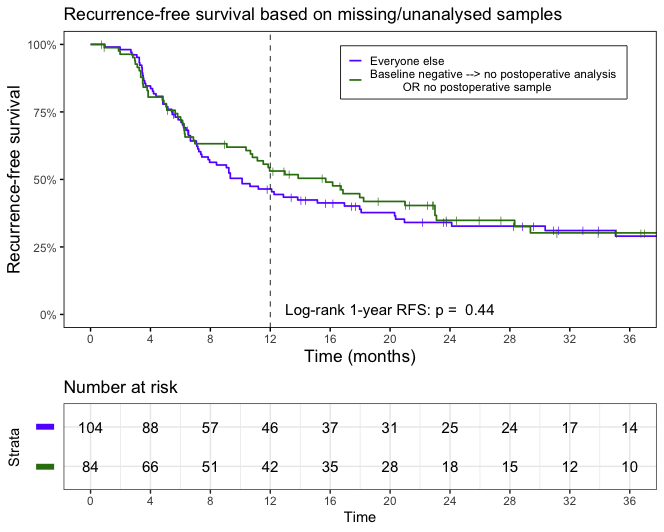


**Suppl Figure 5.** Sensitivity analysis for patients with successful post-surgical circulating tumour cells (CTC) enumeration versus no post-surgical CTC enumeration. No difference in recurrence-free survival (RFS) was observed.

*
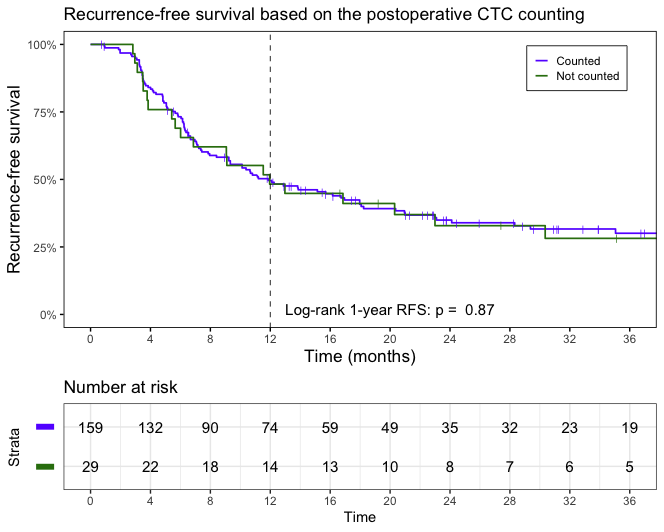
*
