# Supplementary material for: Circulating tumour cells & circulating tumour DNA in patients with resectable colorectal liver metastases (MIRACLE): a prospective, observational biomarker study
Source: eClinicalMedicine. 2025 Aug 12;87:103406. doi: 10.1016/j.eclinm.2025.103406 (PMC12361997; doi:10.1016/j.eclinm.2025.103406)
Supplement: Supplementary_MIRACLE_studyteam [file mmc2.docx]

**List of contributing authours (the MIRACLE study team)**

Ninos Ayez^3^

Jan H. Wijsman^3^

Arjen M. Rijken^3^

Pascal Doornebosch^4^

Joost van der Hoeven^5^

Boris Galjart^1^

Diederik J. Höppener^1^

Peter M.H. Nierop^1^

Eric P. van der Stok^1^

Jean Helmijr^2^

Lindsay Angus^2^

Pauline A.J. Mendelaar^2^

Manouk K. Bos^2^

Elisabeth M. Jongbloed^2^

Khrystany T. Isebia^2^

Noortje Verschoor^2^

Ronald van Marion^6^

Peggy Atmodimedjo^6^

Hendrikus J. Dubbink^6^

Affiliations:

1. Department of Surgical Oncology and Gastrointestinal Surgery, Erasmus MC Cancer Institute, Erasmus University Medical Center, Rotterdam, The Netherlands.
2. Department of Medical Oncology, Erasmus MC Cancer Institute, Erasmus University Medical Center, Rotterdam, The Netherlands.
3. Department of Surgical Oncology and Gastrointestinal Surgery, Amphia Hospital, Breda, the Netherlands
4. Department of Surgical Oncology and Gastrointestinal Surgery, IJsselland Hospital, Capelle aan den IJssel, the Netherlands
5. Department of Surgical Oncology and Gastrointestinal Surgery, Albert Schweitzer Hospital, Dordrecht, the Netherlands
6. Department of Pathology and Clinical Bioinformatics, Erasmus MC Cancer Institute, Erasmus University Medical Center, Rotterdam, The Netherlands.
